# Detection of Pathogens and Regulation of Immunity by the Caenorhabditis elegans Nervous System

**DOI:** 10.1128/mBio.02301-20

**Published:** 2021-03-30

**Authors:** Yiyong Liu, Jingru Sun

**Affiliations:** a Department of Biomedical Sciences, Elson S. Floyd College of Medicine, Washington State University, Spokane, Washington, USA; b Genomics Core, Washington State University, Spokane, Washington, USA; University of Texas Health Science Center at Houston

**Keywords:** *Caenorhabditis elegans*, innate immunity, the nervous system, pathogen recognition, neural regulation, G protein-coupled receptor, neurotransmitter

## Abstract

Although Caenorhabditis elegans has been used as a model host for studying host-pathogen interactions for more than 20 years, the mechanisms by which it identifies pathogens are not well understood. This is largely due to its lack of most known pattern recognition receptors (PRRs) that recognize pathogen-derived molecules. Recent behavioral research in C. elegans indicates that its nervous system plays a major role in microbe sensing. With the increasing integration of neurobiology in immunological research, future studies may find that neuronal detection of pathogens is an integral part of C. elegans-pathogen interactions. Similar to that of mammals, the C. elegans nervous system regulates its immune system to maintain immunological homeostasis. Studies in the nematode have revealed unprecedented details regarding the molecules, cells, and signaling pathways involved in neural regulation of immunity. Notably, some of the studies indicate that some neuroimmune regulatory circuits need not be “activated” by pathogen infection because they are tonically active and that there could be a predetermined set point for internal immunity, around which the nervous system adjusts immune responses to internal or external environmental changes. Here, we review recent progress on the roles of the C. elegans nervous system in pathogen detection and immune regulation. Because of its advantageous characteristics, we expect that the C. elegans model will be critical for deciphering complex neuroimmune signaling mechanisms that integrate and process multiple sensory cues.

## Abstract (precis)

Although Caenorhabditis elegans has been used as a model host for studying host-pathogen interactions for more than 20 years, the mechanisms by which it identifies pathogens are not well understood. This is largely due to its lack of most known pattern recognition receptors (PRRs) that recognize pathogen-derived molecules.

## INTRODUCTION

Although the nervous and immune systems have traditionally been studied in isolation due to the compartmentalization of disciplines, research on neuroimmune communication can be traced back to the 1920s when the study of psychological modulation of immunity using Pavlovian-conditioned stimuli indirectly revealed the existence of interactions between the two systems (1). More recently, work from the last 2 decades of the 20th century uncovered an intricate network of neuroimmune bidirectional communication. Such communication includes, but is not limited to, the production of neuropeptides and neurotransmitters by the immune system that influence the nervous system, the expression of neuroendocrine hormone receptors on immune cells that allows neural signals to influence the immune system, the innervation of lymphoid organs by the sympathetic nervous system, and the regulation of the hypothalamic-pituitary-adrenal (HPA) axis by cytokines (2–4). This wave of discoveries formed the foundation of neuroimmunology, a cross-disciplinary field that specifically studies the various aspects of neuroimmune interactions and has led to the elucidation of basic signaling pathways of communication between the two systems. Despite these advancements, however, many mechanistic details are lacking and remain challenging to study due to the high-degree complexity of the nervous and immune systems in most model organisms (e.g., an adult human brain contains about 86 billion neurons [5]). About a decade ago, several key studies revealed that neuroimmune communication has homologous occurrence in Caenorhabditis elegans, one of the simplest organisms with a nervous system (6–12), indicating that the mechanisms underlying neuroimmune interactions date back to the origins of the nervous system. This conservation paired with the simplicity of its nervous and immune systems makes C. elegans uniquely suited for studying neuroimmune communication in a whole-animal model, and use of this model system has greatly facilitated our understanding of the field.

C. elegans is a 1-millimeter-long nematode worm found in soil and decaying organic matter, where it feeds on bacteria and is inevitably attacked by pathogenic microbes. It does not have an adaptive immune system and relies on innate immunity and avoidance behavior to defend itself against microbial attacks. Upon pathogen infection, the nematode can mount protective responses by triggering evolutionarily conserved immune signaling pathways, including the p38/PMK-1 mitogen-activated protein kinase (MAPK) pathways, the DAF-2/insulin-like receptor pathway, the DBL-1/transforming growth factor β (TGF-β) pathway, the unfolded protein responses (UPRs), and programmed cell death (13). These pathways, while important for fighting infection, must be tightly regulated because insufficient responses can exacerbate infection, whereas excessive responses can lead to prolonged inflammation, tissue damage, or even death (14–19). The C. elegans nervous system plays essential roles in maintaining immunological homeostasis and in the detection of pathogens (20–22). The worm has two sexes, namely, a self-fertilizing hermaphrodite and a male. Hermaphrodites have 302 neurons, while males have 385. The vast majority of research on neuroimmune interactions in C. elegans was done using hermaphrodites because self-fertilization of the hermaphrodite allows for homozygous worms to generate a large number of genetically identical progeny, whereas males arise infrequently and the majority of male-specific neurons are involved in the complex mating behavior (23, 24). The identity, morphology, and synaptic connectivity of each neuron in C. elegans have been well described. The nematode is also the only animal for which a synaptic wiring diagram of the nervous system has been completely established (7). Moreover, most gene families involved in mammalian neuronal functions are found in C. elegans (8). Indeed, the use of C. elegans in studies on neuroimmune interactions has allowed for signaling mechanisms to be dissected at the molecular, neuronal, and organismic levels and has led to the identification of specific molecules, cells, and signaling pathways that regulate host defense (20). Below, we review recent progress on the roles of the C. elegans nervous system in pathogen detection and immune regulation.

## NEURONAL DETECTION OF PATHOGENS

Despite C. elegans having been used as a model host for studying host-pathogen interactions for more than 20 years (21, 22, 25–28), the mechanisms by which it identifies pathogens are not well understood. The nematode does not possess most known pattern recognition receptors (PRRs), which are specialized proteins used by vertebrates and many invertebrate species to detect pathogens through the recognition of pathogen-associated molecular patterns (PAMPs). It also lacks some key components of conserved Toll-like receptor (TLR) pathways, such as NF-κB homologs and the TLR adaptor MyD88 (13). The involvement of PAMPs in triggering the immune response in C. elegans varies greatly among specific pathogens. For example, heat-killed Pseudomonas aeruginosa does not elicit an immune response, whereas heat-killed Staphylococcus aureus does (29). Therefore, how much the nematode relies on PRR-PAMP interactions to identify pathogens remains elusive. Recent studies suggest that C. elegans can sense pathogen attack through the detection of disturbances in cellular homeostasis, a process termed surveillance immunity (30). Similar processes, such as effector-triggered immunity (ETI) and damage-associated molecular pattern (DAMP) response, have been discovered in plants and other animals, respectively (31). For example, upon infection with Pseudomonas aeruginosa, exotoxin A enters the intestinal epithelial cells of the nematode and inhibits protein translation, which triggers the host to upregulate immune response genes (32–34). Disruption of many other cellular activities, such as cell membrane functions, mitochondrial respiration, ubiquitin-proteasome system activity, actin cytoskeleton and microtubule dynamics, and bloating of the intestinal lumen, also results in the activation of detoxification and immune responses (34–40). Indeed, monitoring these physiological changes provides C. elegans with an effective way to identify pathogen attack and launch defense responses.

Although the above-described surveillance immunity allows C. elegans to combat a diverse array of microbes in the absence of a full repertoire of PRRs, defense is triggered only after pathogen attack has occurred and damage has likely already been inflicted, making this strategy, at best, a damage mitigation response. However, is it possible for the nematode to detect pathogens before attack, thus avoiding damage? According to recent behavior research, the answer is yes (21). In fact, pathogen avoidance is an innate skill employed by the tiny worms to survive in the big wild world. For example, C. elegans avoids Bacillus thuringiensis (41) and Microbacterium nematophilum (42) because if exposed, the nematode can be killed or become infected and sick (43–46). Interestingly, the worms also show avoidance behavior to bacteria that are nonpathogenic to them, such as Bacillus anthracis, possibly because they do not like the bacteria as a food source (47). The pathogen-sensing ability of C. elegans is mainly mediated by its nervous system. From a neuroscience perspective, the C. elegans nervous system, like the nervous systems in higher animals that sense and respond to molecular input from the environment, is well suited for pathogen detection. There are 32 chemosensory neurons in the amphid, phasmid, and inner labial organs in C. elegans (48). The amphid, the largest chemosensory organ, is located in the head and is composed of 12 pairs of sensory neurons, of which 11 have cilia exposed to the external environment via openings in the cuticle, giving them direct access to pathogen-derived molecules in the environment (49). Moreover, C. elegans expresses approximately 1,300 G protein-coupled receptors (GPCRs) (20) that may function as chemoreceptors in sensory neurons to detect a diverse array of environmental cues. Chemosensory neurons can directly detect volatile compounds as well as water-soluble molecules. More specifically, AWA, AWB, and AWC olfactory neurons sense volatile odors, and ASE gustatory neurons sense salts and water-soluble attractants (48). For instance, AWC neurons mediate attractive responses to *Pseudomonas* sp.-derived benzaldehyde and isoamyl alcohol, whereas AWB neurons sense repulsive odorants, such as 2-nonanone (50). Bacterial metabolism also generates local fluctuations in gases, such as oxygen and carbon dioxide, and C. elegans can sense and respond to such changes through multiple sensory neurons and GPCRs (21). In addition to the detection of general cues produced by bacteria, the C. elegans nervous system can also sense unique molecules that allow it to distinguish between different pathogens. For example, C. elegans avoids pathogenic Serratia marcescens strain Db10 by sensing the bacterial surfactant serrawettin W2 through the two AWB chemosensory neurons (12). The nematode also escapes from several species of toxin-producing *Streptomyces* by sensing *Streptomyces* sp.-secreted dodecanoic acid through the GPCR SRB-6 and five types of SRB-6-expressing chemosensory neurons (51).

Not all pathogenic bacteria elicit avoidance behavior in C. elegans during their first encounter. Indeed, some bacteria, such as S. marcescens Db11, S. marcescens ATCC 13880, and Pseudomonas aeruginosa PA14, even attract the nematode initially, which could be a strategy developed by the pathogens during the evolutionary host-pathogen arms race to infect hosts (50). C. elegans, on the other hand, can “learn” to avoid these pathogens after its initial exposure, likely due to infection or unfavorable taste, through an experience-based learning response mediated by the nervous system. Such learned aversion is best exemplified by the case of P. aeruginosa PA14 exposure. P. aeruginosa produces a number of virulence factors and can kill C. elegans through multiple mechanisms. In food choice assays, C. elegans initially prefers P. aeruginosa PA14 over its standard laboratory food Escherichia coli OP50. However, after feeding on P. aeruginosa, the nematode subsequently displays a preference for E. coli and an aversion to P. aeruginosa (11, 52). It has been shown that the initial exposure to P. aeruginosa causes an infection that activates strong immune responses (11, 52). Infection also upregulates the expression of *tph-1*, the gene that encodes the rate-limiting enzyme tryptophan hydroxylase in serotonin biosynthesis in ADF chemosensory neurons, which, in turn, enhances serotonin signaling to regulate olfactory learning through the serotonin-gated chloride channel MOD-1 in several olfactory interneurons (11, 52, 53). Meisel et al. (54) observed that exposure to P. aeruginosa, specifically its secondary metabolites phenazine-1-carboxamide and the siderophore pyochelin, activates a conserved G protein-signaling pathway in ASJ chemosensory neurons, which acts cell autonomously to induce expression of the neuromodulator DAF-7/TGF-β in the neurons. DAF-7, in turn, activates a canonical TGF-β signaling pathway through the TGF-β type I receptor DAF-1 in the adjacent RIM/RIC interneurons to modulate aerotaxis behavior and promote avoidance of P. aeruginosa. In a separate study, Singh and Aballay (40) reported that chemosensation of phenazines produced by P. aeruginosa is insufficient to induce pathogen avoidance behavior. Instead, it is P. aeruginosa-induced intestinal infection and bloating of the lumen that triggered avoidance. This effect is mediated by both the DAF-7/TGF-β and the GPCR/NPR-1 signaling pathways. These pathways control aerotaxis behavior, thus promoting aversive learning by changing the worm’s preference from relatively low oxygen lawns of P. aeruginosa to relatively higher oxygen lawns of E. coli. Despite the discrepancies between the above studies, these studies illustrate that both innate and learned avoidance of pathogenic bacteria are mediated by the C. elegans nervous system through sensing pathogen-derived molecular cues in the local environment.

**FIG 1:**
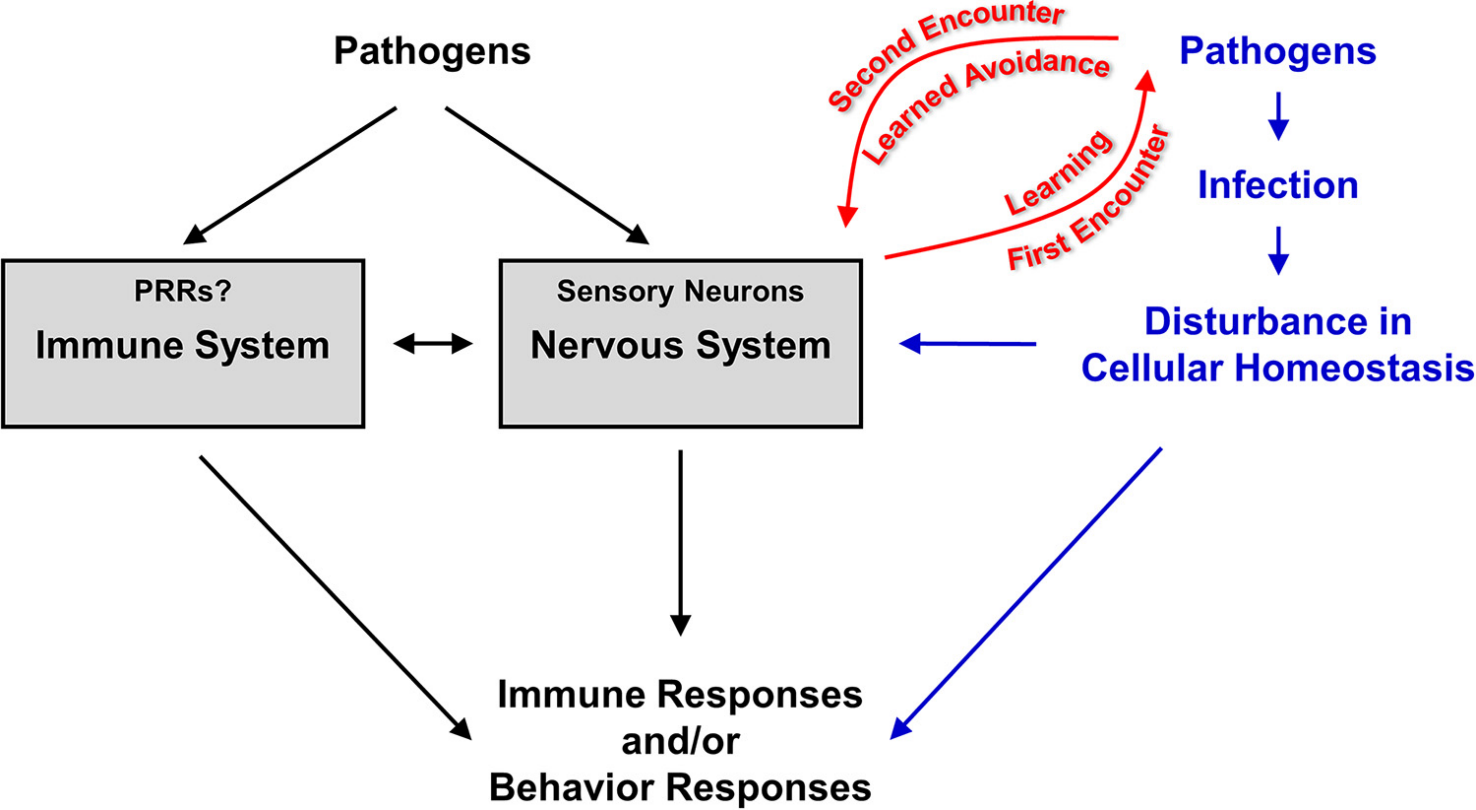
A paradigm of pathogen recognition by C. elegans. Pathogens are detected by either the immune system, the nervous system, or both, which then triggers the innate immune response and/or behavioral responses. Novel pathogens or pathogens that escape detection likely cause infection and disturbance in cellular homeostasis, which subsequently activate immunity and/or behavioral changes. Such infections and disturbances would also signal to the nervous system and trigger learning responses so that the nematode would recognize the same pathogens during a second encounter.

Although the prevailing thinking in immunology is that pathogen recognition is primarily carried out by the innate immune system, the above-described evidence from C. elegans behavioral research indicates that the nervous system also plays a major role in pathogen sensing. Neuronal detection of pathogens is not unique to C. elegans; it is evolutionarily conserved in many animal species, such as Drosophila melanogaster (55) and mammals (56). From an evolutionary perspective, the immune and nervous systems represent analogous evolutionary solutions for animals to sense and respond to internal and external environmental changes. These two systems have such a high level of intersection and share so many similarities in organization and function that it has been proposed that they evolved from a common ancestral cell (57). Pathogen-triggered environmental changes that are sensed by the immune system are likely to have influence on the nervous system and vice versa. In this context and based on the available studies of C. elegans-microbe interactions, it is reasonable to propose that the C. elegans nervous and innate immune systems play similar roles in pathogen recognition. Certainly, it is also possible that the nervous system may be even more important than the innate immune system in this respect due to the innate immune system’s lack of PRRs. The GPCR family is dramatically expanded in C. elegans, with about 7% of all predicted protein-coding genes being GPCRs (58). Most of them (∼1,300) encode nematode-specific chemoreceptors, which can be used by the nervous system to directly sense bacterial compounds, and thus, they could compensate for the absence of PRRs in the C. elegans innate immune system (59). In this paradigm of pathogen recognition by C. elegans, pathogens are detected by either the innate immune system, the nervous system, or both, which then triggers the innate immune response and/or behavioral changes (Fig. 1). Novel pathogens or pathogens that escape detection due to evolutionary selection likely cause infection, accompanied by disturbances in cellular homeostasis, and subsequently activate immunity and/or behavioral responses (Fig. 1). Such infections would then trigger learning responses mediated by the nervous system that would enable the nematode to recognize the same pathogens during a second encounter (Fig. 1). In this paradigm, evidence supporting the neuronal detection of pathogens mainly comes from C. elegans behavioral studies, whereas the number of C. elegans immunity studies in this respect is limited. With the coming revolution integrating neurobiology in immunological thinking (60), future studies may find that neuronal detection of pathogens is an integral part of C. elegans-pathogen interactions.

## NEUROIMMUNE REGULATORY CIRCUITS IN C. ELEGANS

Mammalian studies have revealed that the nervous system regulates the immune system to help maintain immunological homeostasis (14, 15, 61). Early work on nematode neuroimmune communication indicates that such neuroimmune regulatory functions are also conserved in primitive animals. Kawli and Tan (6) showed that defective neurotransmission caused by mutations in *unc-31*, the calcium activator protein required for dense core vesicle (DCV) secretion, increases C. elegans resistance to P. aeruginosa infection and induces the expression of antimicrobial genes, suggesting that neurotransmission from DCVs suppresses innate immunity. Zugasti and Ewbank (62) found that TGF-β signaling from the nervous system promotes the expression of caenacin peptides in the epidermis, which protects animals from infection by Drechmeria coniospora. In addition to these findings, a number of neuroimmune regulatory circuits have been uncovered in C. elegans and are reviewed below.

### The octopamine-OCTR-1 pathway.

OCTR-1, an octopamine GPCR, functions in ASH and ASI sensory neurons to suppress the innate immune response by inhibiting the expression of noncanonical UPR genes of the *pqn/abu* family as well as genes in the p38/PMK-1 MAPK immune pathway (10). These genes are predominantly expressed in pharyngeal and/or intestinal tissues (10, 63–65), indicating that ASH and ASI neurons in the head of C. elegans regulate the innate immune response in distant tissues in a non-cell-autonomous manner. Furthermore, work by Cao et al. (66) found that ASH neurons control innate immunity and that ASI neurons promote pathogen avoidance behavior. They also identified neuropeptide NLP-20 and AIA interneurons as downstream components of the ASH/ASI neural circuit that are responsible for the integration of conflicting cues and behaviors. Although the expression of *pqn/abu* genes fluctuates greatly during development in C. elegans (65), these genes are subject to neural regulation in immune responses to pathogen infection during both developmental and adult stages (67). In contrast, the canonical UPR pathway, which is under the control of X-box binding protein 1 (XBP-1) (68, 69), is regulated by OCTR-1 only at the adult stage and not during development (67). These results indicate that the nervous system temporally controls UPR pathways to maintain endoplasmic reticulum (ER) homeostasis (67). At the protein level, OCTR-1 inhibits specific protein synthesis factors, such as ribosomal protein RPS-1 and translation initiation factor EIF-3.J, to reduce infection-triggered protein synthesis and the UPR (70). Further investigation revealed that octopamine (OA) is an endogenous ligand for OCTR-1 in immune regulation and that the OA-producing RIC neurons function in the OCTR-1 pathway (71). RIC neurons are deactivated by pathogens but are transiently activated by nonpathogenic bacteria. This supports a model whereby an octopaminergic immunoinhibitory pathway is tonically active under normal conditions to maintain immunological homeostasis or suppress unwanted immune responses but is downregulated upon pathogen infection to allow for enhanced immunity (71). Interestingly, the OA-OCTR-1 signaling can be hijacked by bacteria to manipulate host sensory decision-making. The commensal *Providencia* bacteria, which colonize the gut of C. elegans, produce the neuromodulator tyramine, which is converted to OA by the host; the resulting OA, in turn, targets OCTR-1 on ASH neurons to alter aversive olfactory responses (72).

### Neuroimmune signaling via NPY/RFamide-like receptors.

Neuropeptide Y (NPY) receptors are neuronally expressed GPCRs that are involved in modulation of behaviors and immunity (73). In mammals, NPY receptors regulate immunity by suppressing the innate immune system and also by activating antigen-presenting cells (74). C. elegans expresses 41 NPY/RFamide-like receptors (59), among which three (NPR-1, NPR-8, and NPR-9) have been shown to mediate neuroimmune interactions.

### (i) Signaling via NPR-1.

Styer et al. (7) screened 40 worm strains carrying mutations in GPCRs by examining their susceptibility to P. aeruginosa infection and found that a loss-of-function mutation in *npr-1* conferred the worms enhanced susceptibility. Worms harboring mutations in *npr-1* were also found to be more susceptible to two other pathogens, namely, Salmonella enterica and Enterococcus faecalis, indicating that NPR-1 is required for the immune response in general. Mutations in *npr-1* could change oxygen sensation in the nematode, which affects their ability to avoid pathogenic bacteria (75). Experiments under conditions that eliminated pathogen avoidance (e.g., full-lawn assays in which agar plates were completely covered by pathogenic bacteria or low oxygen that suppresses most behavioral phenotypes of *npr-1* mutants) indicated that both altered pathogen avoidance and decreased innate immunity contributed to the enhanced susceptibility to pathogen infection observed (7). Most of the genes that were misregulated in the *npr-1* mutants corresponded to markers of the innate immune response in the p38/PMK-1 MAPK signaling pathway (7). Furthermore, neuron-specific rescue and genetic ablation experiments suggested that NPR-1 functions in AQR, PQR, and URX neurons to control immunity (7). These results revealed an NPR-1-dependent neural circuit that regulates the innate immune response to pathogen infection. Although two neuropeptides, namely, FLP-18 and FLP-21, were identified as ligands of NPR-1 in association with social feeding, behavioral quiescence, and pathogen avoidance (39, 76, 77), the ligand(s) for NPR-1 in the neuroimmune regulatory circuit have yet to be identified, and the signaling details of this circuit remain unknown.

Mutations or polymorphisms in *npr-1* broadly affect C. elegans behaviors, development, and physiology, including susceptibility to pathogens (7, 8, 77–82). Reddy et al. (8) discovered that the pathogen susceptibility difference between the laboratory wild-type strain N2 and the wild isolate CB4856 is caused by a polymorphism in the *npr-1* gene. In agreement with Styer et al. (7), they found that, compared to N2, both the wild isolate CB4856 and animals with a loss-of-function mutation in *npr-1* had enhanced susceptibility to P. aeruginosa. However, they attributed this difference to oxygen-dependent behavioral avoidance rather than direct regulation of innate immunity based on the observations that N2, CB4856, and *npr-1* mutants displayed similar survival against P. aeruginosa under conditions that suppress oxygen-dependent behavioral avoidance, such as full-lawn assays, low-oxygen conditions, and genetic manipulation (8). Despite the discrepancies between the studies of Reddy et al. and Styer et al., these studies suggest that an NPR-1-dependent neural circuit exists in C. elegans to regulate defense responses to pathogen infection either through regulating innate immunity, regulating avoidance behavior, or both.

### (ii) The NPR-8-dependent neural-cuticle defense regulatory circuit.

NPR-8 is another neuronal GPCR in C. elegans that is related to mammalian neuropeptide Y receptors. Functional loss of NPR-8 enhances nematode survival against several pathogens (7, 83), indicating its general role in defense. The improved survival of *npr-8* mutants is not due to changes in pathogen avoidance behavior, pathogen intake, or conserved innate immune signaling pathways (83). Instead, transcriptome sequencing (RNA-seq) analyses and functional assays have revealed that NPR-8 regulates C. elegans defense by suppressing the expression of cuticular collagen genes and by controlling the dynamics of cuticle structure. The defense activity of NPR-8 is confined to the amphid sensory neurons AWB, ASJ, and AWC. Thus, an NPR-8-dependent neural-cuticle defense regulatory circuit has emerged, whereby NPR-8 functions in AWB, ASJ, and AWC neurons to regulate cuticle dynamics by suppressing the expression of cuticular collagen genes, which, in turn, negatively influences the nematode’s defense against infection. The cuticle and collagens have essential structural roles in barrier defense against pathogen infection and other environmental assaults (84). Interestingly, a recent study found that the dual oxidase CeDuox1/BLI-3 functions in both innate immune defense and collagen cross-linking required for cuticle integrity, but these two functions are unrelated to each other (85). Because the cuticle is not innervated, the NPR-8-dependent neural-defense regulation is likely achieved through neuroendocrine signaling. However, the details of such non-cell-autonomous regulation are currently unknown.

### (iii) Signaling via NPR-9.

NPR-9, a nematode homologue of mammalian gastrin-releasing peptide receptor (GRPR), modulates multiple biological processes in C. elegans, including fat storage and local search behavior and is exclusively expressed in AIB interneurons (86). Yu et al. (87) found that a loss-of-function mutation in *npr-9* confers C. elegans increased resistance to P. aeruginosa infection, which is not attributable to alternations in fitness but due to enhanced innate immunity. Expression of *npr-9* under its own promoter, presumably in AIB interneurons, rescued the increased resistance phenotype of the mutants, while overexpression of *npr-9* decreased the resistance to below the wild-type level, suggesting that NPR-9 suppresses the innate immune response to P. aeruginosa. Interestingly, activation of AIB neurons either by optogenetic manipulations or by expression of *pkc-1(gf)*, the active protein kinase C homologue increased the immune response and resistance against P. aeruginosa. Expression of *npr-9* under these conditions suppresses the increased resistance, indicating that NPR-9 can antagonize the activity of AIB interneurons in immune regulation. It is not clear why there are conflicting immunoregulatory pathways in a single type of neurons and how these pathways interact to influence the overall functional output of immune responses to pathogen infection.

### The serotonergic pathways.

As described above, serotonin signaling mediates learned avoidance behavior in C. elegans (11, 52). Exposure to P. aeruginosa causes infection, which increases serotonin synthesis in ADF chemosensory neurons and promotes aversive learning so that the animals alter their olfactory preferences to avoid these bacteria in future encounters (11, 52). In mammals, serotonin regulates both innate and adaptive immunity (88, 89). A recent study by Anderson et al. (46) found that serotonin signaling similarly controls immunity in C. elegans against the naturally occurring bacterial pathogen *M. nematophilum*, suggesting that the immunoregulatory function of serotonin is evolutionary conserved. Upon infection with *M. nematophilum*, C. elegans displays swelling around the rectal opening, termed the deformed anal region (Dar) phenotype, which is a hallmark of the immune response triggered by this pathogen (44–46). Both exogenous serotonin and endogenous serotonin synthesized by TPH-1 in ADF chemosensory neurons suppress the Dar phenotype (46). Two GPCRs for serotonin, namely, SER-1 and SER-7, are required for the negative regulatory effect of serotonin on the C. elegans immune response to *M. nematophilum* (46). Moreover, this regulation also requires conserved G-protein signaling through GOA-1(Gαo) in rectal epithelial cells (46). It is not clear how ADF neurons in the animal’s head signal to the distant rectal epithelial cells in the animal’s tail to regulate immunity.

### The dopaminergic pathways.

The neurotransmitter dopamine (DA) modulates a number of key functions, such as behavior, voluntary movement, feeding, attention, affect and motivation, pleasure and reward, and drug addiction (90). In mammals, it regulates the innate immune response as well as the adaptive immune response particularly T lymphocytes (90). In C. elegans, Anyanful et al. (9) found that brief pre-exposure of animals to the lethal bacterial pathogen enteropathogenic E. coli (EPEC) increased their survival upon a subsequent exposure, a phenomenon that the authors termed “conditioning” (9). This effect was determined to be dependent on the p38/PMK-1 MAPK and the insulin/IGFR pathways through the regulation of immune and life span genes, respectively. Animals with defective neurotransmission were found to not exhibit conditioning, indicating that conditioning is mediated via neurotransmission. Further analyses using a variety of mutant animals and chemical-mediated killing of neurons showed that dopaminergic signaling, including dopaminergic neurons and DA receptors, but not serotonergic signaling, is required for conditioning. These findings suggest that the nervous system can adaptatively regulate the immune response based on how or how much the host is exposed to pathogens.

In a separate study on dopaminergic immunoregulation, Cao and Aballay (91) reported that treating C. elegans with the DA antagonist chlorpromazine enhanced its resistance to P. aeruginosa infection by activating the p38/PMK-1 MAPK immune pathway. These results suggest that dopaminergic signaling suppresses the innate immune response to P. aeruginosa. Among the four DA receptors examined (DOP-1, -2, -3, and -4), only *dop-4* mutants showed the enhanced resistance phenotype, indicating the involvement of DOP-4 in the suppression of immunity. Neuron ablation and rescue experiments identified the dopamine-expressing CEP neurons and the DOP-4-expressing ASG neurons as the necessary neurons for the immune regulation. These results suggest that upon binding DA released from CEP neurons, DOP-4 functions in ASG neurons to control the immune response to pathogen infection.

### The cholinergic pathways.

Acetylcholine (ACh) is a ubiquitous signaling molecule that regulates numerous biological processes. In many animal species, ACh is produced by both neurons and nonneuronal cells, including immune cells, and as such, it is a major mediator of neuroimmune communication (92). In C. elegans, ACh is produced only in the nervous system (93) and is involved in modulating both avoidance behavior and the innate immune response (94, 95). McMullan et al. (94) showed that C. elegans avoids *M. nematophilum*, but such aversion behavior was lost in animals with mutations in *egl-30*, the gene that encodes the α subunit of heterotrimeric G protein q (Gαq). Expression of *egl-30* in cholinergic motor neurons partially rescued the aversion behavior of the *egl-30* mutants, suggesting that cholinergic neurons modulate the aversion response.

A recent study by Labed et al. (95) demonstrated that cholinergic signaling also regulates the innate immune response in the intestinal epithelium of C. elegans against Staphylococcus aureus infection. To search for GPCRs that may detect S. aureus infection, the authors screened 890 GPCR genes using RNA interference and found that silencing *gar-2* and *gar-3*, the genes that encode muscarinic ACh receptors, suppressed the expression of S. aureus-induced immune genes. Moreover, treatment with the ACh-mimic arecoline or muscarinic agonist oxotremorine induced the expression of immune genes, which can be abrogated by silencing *gar-2* or *gar-3*, whereas treatment with muscarinic antagonist scopolamine impaired immune gene induction by either S. aureus or arecoline, indicating that cholinergic signaling is necessary and sufficient to mediate the innate immune response. By using an array of genetic, chemical, and imaging techniques, the authors delineated this signaling pathway in detail, as follows. S. aureus infection causes the nervous system to release ACh, which then activates muscarinic receptors in the intestinal epithelium in a neuroendocrine manner; this activation triggers transcription factors such as LIN-1 to increase the expression of Wnt and its receptor Frizzled, which leads to the induction of immune genes.

### Neuroimmune signaling via neuronal protein OLRN-1.

Foster et al. (18) conducted a forward genetic screen to identify endogenous regulators of the p38/PMK-1 MAPK pathway and found that mutations in *olrn-1* caused constitutive activation of this pathway. OLRN-1 is a neuronally expressed protein that controls the expression of olfactory receptors in AWC chemosensory neurons during development (96). *olrn-1* null animals displayed enhanced resistance to P. aeruginosa infection as well as increased transcription of immune effector genes in the intestine. Expression of *olrn-1* in neurons, especially in AWC neurons, fully rescued the enhanced resistance phenotype of *olrn-1* null animals, suggesting that OLRN-1 functions in chemosensory neurons to regulate innate immunity in the intestine in a non-cell-autonomous manner. These data established a connection between OLRN-1-mediated neural control of the p38/PMK-1 MAPK pathway and olfactory receptor development, indicating significant roles of neuroimmune regulation in nematode development and physiology.

## CONCLUDING REMARKS

Although mammalian studies on neuroimmune communication started earlier than studies in C. elegans, the latter have revealed unprecedented details regarding the molecules, cells, and signaling pathways involved in neuroimmune regulation. Based on mammalian studies, specifically the stimulation of cholinergic vagal nerves to suppress the production of TNF, Tracey proposed the inflammatory reflex theory, which posits that inflammatory stimuli activate neural circuits which, in turn, trigger anti-inflammatory responses (61). Results from studies in C. elegans are largely in agreement with this theory. However, the C. elegans studies also indicate that some neuroimmune regulatory circuits need not be activated by pathogen infection because they are tonically active to maintain immunological homeostasis. This idea is supported by the observation that inactivation of such circuits under normal, noninfectious conditions results in a higher expression of immune genes (6, 18, 71, 91). This notion is also consistent with clinical evidence showing that decreased or severely impaired anti-inflammatory neural circuits are the major underlying cause for many innate immune diseases, such as sepsis, arthritis, inflammatory bowel disease, and hemorrhagic shock (97). Based on these studies, it is reasonable to speculate that there is a predetermined set point for internal immunity, like the set point for human heart rate, and that the nervous system reflexively regulates immune responses to internal or external environmental changes and restores immune homeostasis by bringing the immunity back to the set point (60). This speculation is substantiated by experimental evidence from studies in C. elegans suggesting that neural signaling that regulates immunity can be either inhibitory or excitatory (98) and that the extent of regulation is calibrated based on what bacteria are encountered (71) or how they are encountered (9). These findings have great clinical potential because neural circuits could be either stimulated or inhibited electrically or pharmacologically to maintain or restore internal immunological homeostasis.

Neuroimmune communication is a complex biological process. In nature, animals often encounter multiple environmental cues that are simultaneously present, such as food availability, pathogenicity, temperature, and odors. The functional output of neuroimmune interactions is likely the net result of multiple regulatory circuits that integrate and process these cues. With current technology, it is difficult to mechanistically understand humans’ conscious decision to “drink poison to quench thirst.” However, characteristics of C. elegans, such as the simplicity of the nervous and immune systems, genetic tractability, effectiveness of RNA interference and their transparent body that allows for monitoring gene expression, could enable us to decipher the worm’s unconscious choice between their last meal of pathogenic bacteria and starvation. From this point of view, the C. elegans model will be critical for understanding complex neuroimmune signaling mechanisms that integrate and process multiple sensory cues.
